# Old Way and the New in Dental Practice

**Published:** 1874-02

**Authors:** S. Welchens

**Affiliations:** Lancaster.


### ARTICLE IV.

### *Old Way and the New in Dental Practice.*

BY S. WELCHENS, D. D. S., LANCASTER.

In these days of scientific wonder and rapid professional development, it may be well to take a retrospective view of our specialty, and thus endeavor to improve the future through and by the experience of the past. It will doubtless be conceded by all practicing dentists, who are capable of ordinary observation, that there has been, in the last three or four years, so much of a change in the operative branch of the dental profession as to almost amount to a complete revolution. The old method of health and usefulness, has given way to a system far superior and almost diametrically opposite, through a succession of new discoveries, appliances, improved material and scientific treatment, to such an extent, as to make the expert in the old way, who has not kept up with the times, a perfect novice in the new; and those who are regarded as first-class operators in the new way, who have become so alone by virtue of these improvements or appliances, to be unable to operate with any degree of success or merit in the old.

Now, whilst we regard it as a duty as well as a pleasure to keep pace with and encourage meritorious advancement, and every new and valuable discovery, we nevertheless deprecate, to some extent at least, the formation of "*corners*," by which old, and what in the days of Harris were regarded as scientific operations, are hid out of sight.

For instance, here is an old practitioner, enjoying both the confidence of his profession and the community in which he lives. He has kept up with the age of improvement and abreast with best operators for a quarter of a century; whilst round the "*corner*" is a young man with but four or five years' experience, regarding him as an "old fogie," and characterizes his best work as mere quackery, simply because he was educated in the old principles, and still adheres to some of the old practices in operating. We must confess that there is something about this that does not meet our ideas of what might be regarded as scientific professional advancement. It ignores experience and wipes away well established principles which are the result of years of meritorious practice.

It is unquestionably the duty of a practitioner to avail himself of every principle and appliance that will improve his operations and enhance his knowledge of the complications of his profession. But at the same time, a rational scientist will respect old practices that were good in their day, and in his researches will seek a sort of "*ex-post-facto*" training before arrogating to himself leadership, or absorbing within himself all that is really meritorious in his profession. Instead, therefore, of an abrupt "*corner*" being formed that might hide from the view of our young and conceited members of the profession, those old principles which brought it up to a respectable standard in the scientific world in former years, there should be a blending of the elements of improvement; a sort of succession of links, as it were; or a "mathematical progression," if you please, fortifying the new with the old, thus connecting two systems in one grand comprehensive status, in which the operator may be made to feel at home in good practice, and which would afford him all the resources necessary to meet new and complicated cases without drawing on other men's brains, genius or experience when difficulties arise. His education should enable him to be "eclectic" at times, which simply means to let in the light of other days and the old authors; and not despise the labor and the genius that laid the foundation for the improvements we are now enjoying.

*Improvements in Dental Depots.*—Everything that will save time and labor is a real, substantial benefit and aid to the dentist. There was a time when dental depots were both scarce and poor. They, like the profession, rejoiced in the magnificence of their professional meagreness. Then, while the depots were poor, there was some danger of the dentists becoming rich. But since the extreme prosperity of the former, the best efforts of the latter to reach that devoutly wished for consummation is fruitless.

We do not complain of the commendable energy exercised by the depots in emulating each other in getting up new appliances, and surrounding each new discovery with so much interest and real benefit to the dentist as to render it absolutely necessary that he should avail himself of the improvement, or fall ten years behind the times. This, 'tis true, is a diversion which turns the tide of prosperity from the profession toward the manufacturers. But, then, this enterprise has given us so much that is really good that we can afford to work "from hand to mouth," to keep it up, and the fact that it averts the danger of our ever becoming rich should be sufficient to reconcile any dentist to the change it has produced.

For example, twenty-five years ago, when I purchased my "*kit*," it took me three or four days to gather up the "*traps*," and spend my hundred dollars. That sum was to me then a small fortune, and it seemed to be about the same thing to the "*depot*," for it did not only secure me the most unremitting attention, but it fairly exhausted the depot. Now you can spend that sum in your office some fine morning before breakfast, (provided the itinerant depot vender stops over night in your town,) and get for your money the half of a Harris' chair, or two-thirds of a dental cabinet, or one Morrison engine and the half of a fountain spittoon, or an electric plugger and patent stool; all as specimens of the latest improvement, and the most trifling purchases. *Then* the selection of a set of teeth to suit your case would require a whole day, provided you had a good assortment to select from. Teeth were measured out to you by the quart, and if the "assortment" was good, of course the *mixture* would be grand; and the whole day thus consumed was put in with the most exquisite professional torture. When, therefore, artificial teeth came to us arranged in sets, and spread out on strips of wax, the change was almost overwhelming, and the improvement was of incalculable value.

Thus the depots have been creeping up, making valuable improvements; adding one new appliance to another—one useful discovery to another, until we are dazed with the beauty and grandeur of the display, and feel that we are really ten years behind the age of improvement when we see their stock of goods, and feel unable to try every new thing, or appropriate the entire selection. That such enterprises should reap the reward of colossal fortunes is not to be wondered at, and ought not to be begrudged.

*Improvements in Mechanical Dentistry.*—There was a time when artificial dentures possessed real intrinsic value. They were mounted upon gold and and silver plate, and regarded by the wearer as perfectly comfortable and serviceable. In those primitive times, plain plate teeth were used, and mounted on plates that were cut out so as to be simply wide enough to cover the ridge of the maxillary jaw, Where an entire denture was needed, spiral springs were used to keep the plates firm to the gum.

This work, though good in its day, was somewhat troublesome, both to the dentist and the patient. Wax being used for impressions, the fit, of course, was approximate, and the benefit to the patient about the same way, until custom overcame the difficulty. At a subsequent period, however, improvements were introduced, and with the advent of the air chamber and gum teeth, we imagined the advancement a little too rapid, and readily consented to stop a while to enable all to get up with the times. Rims on the plates, both swaged and turned down, were added to the general tenure of this work, and block teeth, continuous gum work, and the various methods of soldering to avoid warping, were among the more immediate improvements.

The laboratory became the centre of science in the profession, and our best men emulated each other in raising this branch of the profession to the highest standard of artistic skill. The work produced by our best operators was really beautiful and perfectly serviceable. It was science in art, and constituted an era in the profession that should not only not be despised, but should never have been suffered to be superseded. The downfall of this art started with the advent of casting plates from the baser metal, and the use of rubber and other vegetable substances. Beautiful and even serviceable dentures were, and still are constructed from those cheap materials; but the change was so great, and the manner of performing the work so opposite, that a "*corner*" was formed, rendering those who are taught and skilled in the latter mode of mounting teeth alone, unable to work in the former with any degree of success.

The very simplicity of this latter kind of work, and the ease with which it was constructed, drove science out of the laboratory, and reduced the mechanical branch of the profession to the level of a trade or handicraft. So far, therefore, as the improvements in this department are concerned, the progress has been backwards from a scientific standpoint, and it behooves us all to see to it, that the operative branch through this progression of appliances and new discoveries does not meet with the same disaster, thus making the last great blunder worse than the first. We think we see symptoms of this already in the increase of the use of the baser metal in foils and amalgams, and the various cements and stuffings which are too readily resorted to where a more complicated and more permanent operation should be performed. Is there not some cause here to fear that that nice artistic manipulation of pure gold will be forced to surrender to the overpowering advance of those cheaper materials in filling teeth?

*Improvements in Operative Dentistry.*—The new mode of operating with the appliances now in vogue, leads the mind into a similar channel of thought, with well grounded fears for the future in that department of our specialty.

With the rubber dam, burring engines and improved patent pluggers of recent date; it does not require much more talent or skill to become a good, even a first-rate operator in filling teeth, than it did several years ago to put up first-class dentures on the rubber base. The most difficult part of the operation to manage is to adjust the rubber dam. When that is properly done, an ordinary individual, with but a low degree of mechanical ingenuity and but partially expert in working gold, can perform very difficult operations and bring them up to what is termed a first-class standard, when the same parties could with difficulty reach mediocrity in the old method of practice.

Here then is a fruitful source of solicitude, lest what we now look upon as the highest attainments of skill be lowered both in value and excellence, by comparatively ignorant and irresponsible men.

It is not our purpose to disparage those improvements, or to underrate those operations which are so well performed by scientific men. But is it not apparent that since the profession has been benefitted by those appliances, what may be termed good operators are far more numerous, whilst earnest, thinking, reading and scientific men are rather on the decline?

But it may be asked, what have we to fear from all this? Why just this. The profession will be literally run down with good superficial operators, who care more for their own pockets than the status of the profession, and it will be dragged down to the level of a common mechanical pursuit. Or, in other words, the mechanical element will be highly cultivated, and the competition arising therefrom, as in the rubber work, will reduce the valuation of such work, the intellectual element will become stagnant, the qualifications of the dentist will be meagre and low and unable to reach the true genius of the profession as a branch of the healing art.

As a profession, we want first-class operators. We also need all the appliances we can get to facilitate our operations; but at the same time we must give heed to and appreciate old and well-tried principles, and thus guard and develop that balancing intellectual power which gives the mind scope and discrimination to comprehend the complications of disease, so as to be able fully to meet every case with a due sense of that responsibility which should characterize a member of an honorable profession.

This, of course, will admit of no cutting away of old principles, or even the old method of practice, so as to form a "corner." But it will call into requisition every element of improvement, and lead men to value more highly those primitive principles which served as stepping-stones to higher and more advanced stages of excellence.

There is a majesty in the moral force of a scientific profession that should be the chief safeguard to its dignity and usefulness, and that should dictate the rule of action in the case of any man who would excel as a practitioner. This does not consist alone in the superiority of this or that operator's work—his own sense of his own excellence, nor of the amount of "*blowing*" he may be capable of. It is the force and power of the quality of usefulness he may possess, in rendering himself useful to the public, with a reciprocal appreciation, and without any further effort to please than simply to *perform well a present duty*, rather than that any man should indulge in this preposterous self-laudation.

The dental profession, like the medical profession, is an untiring and ceaseless research after *truth*. In every instance where pathological conditions are present, and their locality and extent are determined by symptoms alone, every step and movement made in the diagnosis is a search for the truth. If, therefore, there is a deficiency here, and the individual who adopts our profession is superficial in those fundamental principles, no matter how meritorious his mechanical manipulations may be, *he is a quack*, and no amount of good work or self-praise will extricate him from that unenviable position.

This idea, then, is all that is left to connect the old way with the new. It is not our operations alone, for in both branches of the profession they are performed entirely different to what they were ten years ago. It is the practitioner's scientific calibre; his skill in the healing art; his knowledge of the principles and practice, old and new; his theoretical research, and his ability to grasp and comprehend every case, however complicated; his correctness of judgment, and his boldness to execute these render him the reliable, skillful operator, and master of his science.—*Transactions of Penn. State Dental Society.*

